# Analysis of the Frequency of Acoustic Emission Events in Terms of the Assessment of the Reduction of Mechanical Parameters of Cellulose–Cement Composites

**DOI:** 10.3390/ma14195882

**Published:** 2021-10-08

**Authors:** Anna Adamczak-Bugno, Aleksandra Krampikowska, Grzegorz Świt

**Affiliations:** Faculty of Civil Engineering and Architecture, Kielce University of Technology, 25-314 Kielce, Poland; akramp@tu.kielce.pl (A.K.); gswit@tu.kielce.pl (G.Ś.)

**Keywords:** cement–cellulose composites, ventilated façade, acoustic emission method, frequencies of acoustic emission signals

## Abstract

The article proposes the application of the acoustic emission method as a technique for the evaluation of mechanical parameters of cellulose–cement composites. The analysis focused on frequency values in a time series analysis of elements subject to three-point flexural stress. In the course of a statistic analysis, it has been demonstrated that a significant reduction of the recorded frequency values is associated with a considerable reduction in strength. This allowed the authors to determine the range of frequencies related to the depreciation in the strength of an element. The tests were carried out on elements cut from a full-size cellulose–cement board. Samples exposed to potential operational factors (environmental and exceptional) were analysed. It was shown that the frequencies recorded before reaching the maximum load during bending of samples exposed to environmental factors (water and low temperature) were significantly different (were much lower) from the sounds emitted by elements subjected to exceptional factors (fire and high temperature). Considering the fact that the analysed frequencies of acoustic emission events occur before the maximum stresses in the material are reached and the elements are destroyed, this provides the basis for the use of the acoustic emission method to assess the condition of cellulose–cement composites in terms of lowering mechanical parameters by observing the frequency of events generated by the material during load action. It was found that generating by material frequencies above 300 kHz during bending does not result in a significant decrease in mechanical parameters. The emission of signals with frequencies ranging from 200 to 300 kHz indicate that there was a decline in strength exceeding 25% but less than 50%. The registration of signals with frequencies below 200 kHz indicates that the reduction in mechanical parameters was greater than 50%.

## 1. Introduction

Building materials produced on the basis of cement reinforced with organic fibres have been used in the building industry for over a century. It is believed that this type of material was invented by Ludwik Hatschek, a Czech engineer who patented the methodology of manufacturing panels known as ‘Eternit’. Cement elements with an additive of asbestos fibres exhibited relatively high strength parameters, while also being non-absorbent and non-flammable [1,2,3,4,5,6,7,8]. Those very features made Eternit one of the most popular types of roofing in the 1980s and 1990s. Their popularity decreased when it was revealed that asbestos fibres have carcinogenic properties. From then on, efforts have been undertaken to develop a replacement technology. Over the course of the performed research, cement matrices were strengthened using various types of organic fibres, variable, e.g., by origin or length [9,10,11,12].

One of the most common fibrous cement composite products are panels based on a cement matrix reinforced with cellulose fibres. These panels, apart from Portland cement and organic fibres, can also contain synthetic fibres as well as special additives or ad-mixtures, which improve their strength and performance parameters [13,14,15,16,17].

Most cellulose–cement panels are designed for internal and external use (e.g., as siding elements in ventilated facade systems). Due to working conditions present in the case of their external use, as facade elements, it is necessary to determine the effect of any potential operating factors on the parameters of the boards. Many literature sources cite examples that demonstrate the deterioration of aesthetics as well as of the mechanical parameters of the panels due to the impact of external conditions [18,19,20,21,22]. Due to the fact that cellulose–cement panels are currently also used in buildings classified as tall buildings and high-rise buildings, for safety reasons, it is necessary to develop a method which would facilitate the determination of the actual condition of the panels during their routine inspections. This would help to eliminate any possibility of façade siding elements falling down from the building, resulting in personal and material hazards [23,24,25,26,27,28].

The operating conditions of cellulose–cement panels primarily include the effects of water and moisture, resulting in the cyclical soaking and drying of the panels, as well as the regular changes between above-zero and sub-zero temperatures. In addition, when analysing the service life of the boards, the possible occurrence of exceptional conditions must be taken into consideration, among which the effects of fire and high temperatures seem the most destructive [29,30,31,32,33,34].

Considering that cellulose–cement panels must be inspected at the site of their installation, it is necessary to develop a non-destructive methodology that would enable their monitoring, preferably remotely. The acoustic emission method is the one which is becoming increasingly popular when used to this end, as it allows for the observation of load-related active destructive processes in materials. Acoustic emission has been successfully implemented as a research methodology for the diagnosis of engineering structures made of concrete and steel. The results of tests performed on cellulose–cement panels have also been documented in literature. Previous analyses were performed using neural networks as well as the acoustic emission method, with the application of the energy-related and time/frequency-related approach [35,36,37,38,39,40].

The implementation of the abovementioned models in the context of the acoustic emission method requires the performance of analyses using specialised software, the so-called artificial intelligence, to facilitate the learning of neural networks, the classification of AE signals, as well as the performance of a time and frequency analysis. However, based on the performed research, the authors have observed that the frequency of events is one of the most variable parameters of acoustic emission, sensitive to changes in the mechanical parameters of a material. Therefore, they performed an analysis that allowed them to determine the significance of these changes and to associate them with a specific degree of reduction of the strength of cellulose–cement elements [41,42,43].

Testing the dynamic parameters of composites is an issue widely described in the literature in relation to composite layered structures [44] and GFRP composites [45]. Due to the use of modern research techniques and computer methods, it is possible to accurately assess the influence of geometric parameters of an element on mechanical indicators both in static and fatigue tests [44,45].

The basic research question posed by the authors concerned checking whether the frequencies of acoustic emission events are related to the mechanical parameters of composites, and if so, whether the relationship between them is statistically significant. The next question was to check how the potential operating conditions affect the mechanical parameters and frequency of acoustic emission events.

The authors decided to observe the frequency of AE events emitted by cellulose-cement composites because in other publications, this parameter allowed for the tracking of changes in mechanical parameters in brittle materials [46,47].

The novelty of the research carried out consists in the observation of one selected energetic parameter of the acoustic emission. In previous publications on cellulose–cement composites, the use of the AE method was associated with the procedure of teaching neural networks or unsupervised signal classification, which requires specialised software and skills. The AE event frequency analysis can be performed immediately after the measurement and even during the measurement without the need to transfer data, which significantly extends the availability of the acoustic emission method as a tool for assessing changes in mechanical parameters in cellulose–cement composites.

The main benefit of the present research study for researchers and users concerns the ability to refer, when assessing the mechanical parameters by observing the frequency of AE events, to the results obtained for samples of cement–cellulose composites exposed to potential operational factors. The presented results and the criteria proposed in the conclusion were also confirmed during other analyses, which concerned the classification of 14 parameters of AE events, the observation of time–frequency spectra, and the study of the microstructure of the material. According to the authors, the proposed research procedure is the basis for the creation of simplified guidelines enabling the inspection of the condition of cellulose–cement composites without the need to use specialised analysis software.

## 2. Materials and Methods

**Figure 1:**
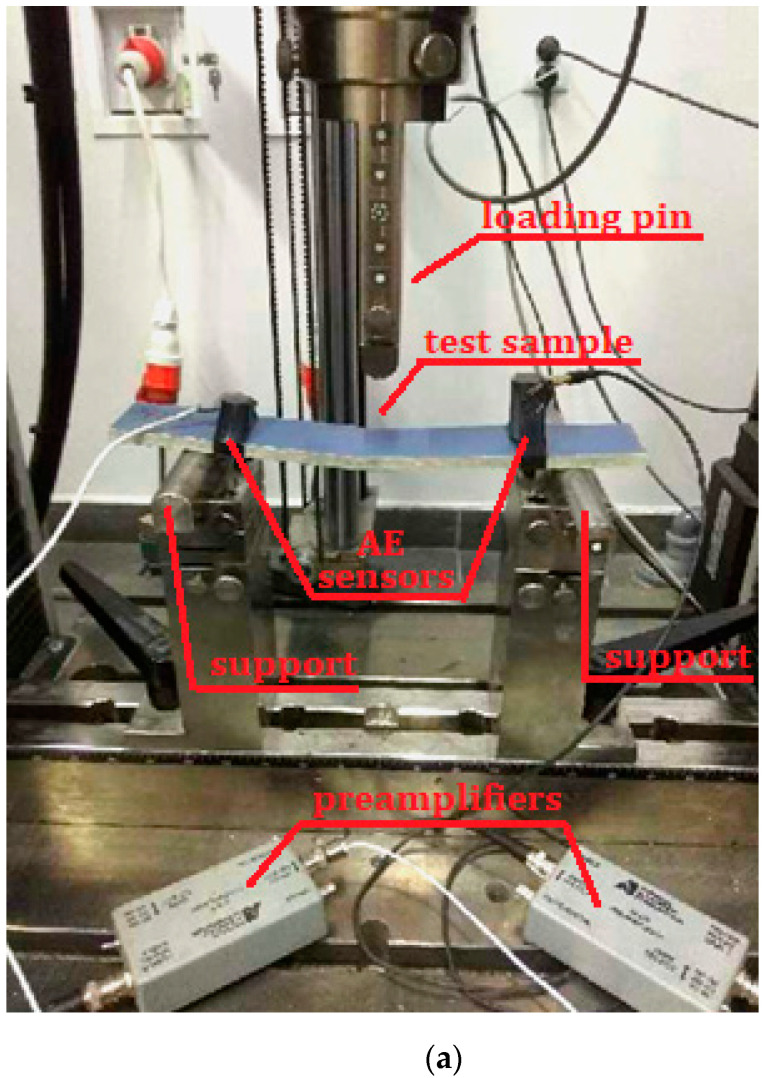

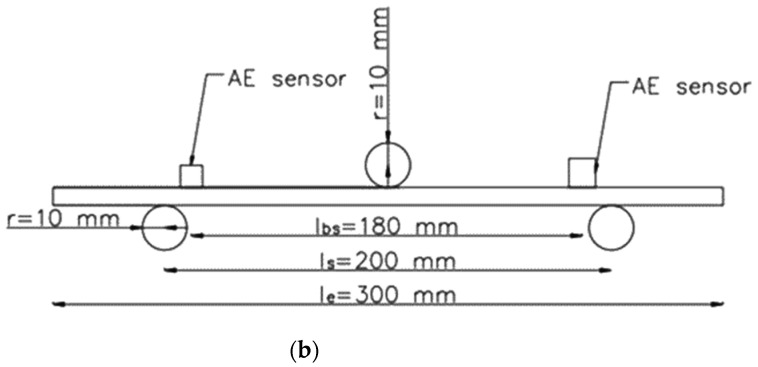
Test stand diagram: (**a**) a photograph of one of the samples and (**b**) the load diagram.

The tests were performed on elements cut out of full-size cellulose–cement panels. Cuboidal samples of the dimensions of 300 × 50 × 8 m^3^ were cut out of stock panels of the dimensions of 1.25 × 3.10 m^2^, 8 mm in thickness. The samples were cut parallel to the length of the panels. A 50 mm marginal zone of the panels was omitted during preparatory work. A view of the test stand is shown in Figure 1.

Following the tests, frequency values in the analysed time series for the tested elements were monitored. In addition, changes of the loading force *F* in time were also observed.

On the basis of the completed preliminary tests, it was confirmed that cellulose–cement panels demonstrated the mechanical parameters declared by the manufacturer. The chemical formula of the panels and the details of their technological process are patent-protected, and any information referring to the specific components, their quantities and suppliers, as well as any production details are very restricted. According to the manufacturer’s declaration, the tested fibre-cement panels were made using basic components such as: Portland cement CEM I 42.5N, cellulose fibres, or PVA synthetic fibres. The elements also contained an additive in the form of lime powder. The manufacturing process of the panels was based on Hatschek’s process. The scope of application of the panels was declared by the manufacturer as indoor and outdoor. The average technical parameters declared by the manufacturer of the panels are included in Table 1.

The tests were performed on cellulose–cement panels operating in conditions of an environmental and exceptional nature. The following research cases have been identified:

- air-dry condition;
- saturation with water for 1 h;
- saturation with water for 24 h;
- 25 bathing–drying cycles;
- 50 bathing–drying cycles;
- 10 freezing–unfreezing cycles;
- 25 freezing–unfreezing cycles;
- 50 freezing–unfreezing cycles;
- 100 freezing–unfreezing cycles;
- direct contact with a flame for 2.5 min;
- direct contact with a flame for 5 min;
- direct contact with a flame for 7.5 min;
- direct contact with a flame for 10 min; and
- contact with the temperature of 230 °C for 3 h.

Panels in the first case P_1_—the reference case—were stored in the conditions of constant temperature and humidity (+23 °C, 60% humidity). This case was considered as a benchmark.

Samples from series P_2_ ÷ P_3_ were submerged in water of room temperature (approximately 23 °C) for a period of 1 and 24 h, upon which they were subjected to wet flexure tests.

Bathing and drying cycles (cases P_4_ ÷ P_5_) were performed by alternately submerging the samples in water of an ambient temperature higher than 5 °C (approximately 23 °C) for 18 h and drying them in a ventilated drier at a temperature of 60 °C (±5 °C) and relative humidity lower than 20% for 6 h; the number of cycles depended on the research case (P_4_ —25 cycles; P_5_—50 cycles).

Cyclical freezing and unfreezing (cases P_6_ ÷ P_9_) was performed in a specific air-water environment via alternate cooling (freezing) in a freezer, in a temperature of −20 °C (±2 °C) for 2 h and this temperature was maintained for another hour, followed by subsequent heating (unfreezing) in a water bath at a temperature of 20 °C (±2 °C) for two hours and this temperature was maintained for another hour. During the cooling and heating cycles (freezing and unfreezing), the samples were positioned in a manner that ensured free circulation of the conductive medium (air in the freezer or water in the bath).

The baking of the fibre-cement panels took place in a laboratory oven (Kedndrolab, Warsaw, Poland) at a temperature of 230 °C. The duration of the baking was 3 h, which led to the total destruction of the fibres in the material.

The impact of fire is an exceptional factor that involves exposure to high temperatures which occur in the case of events such as a building fire. The process of the destruction of fibre-cement panels involved the direct application of a flame, resulting in the surface temperature of the panel reaching approximately 400 °C for a time of 2.5 to 10 min, recorded at 2.5-min intervals (cases P_10_ ÷ P_13_).

Table 2 presents a list of test cases of cellulose–cement composites with the adopted sample designation.

Each research case included 10 samples. The static scheme and the dimensional proportions of the samples were adopted in accordance with [48], product specification and test methods.

Flexural tests of cement-fibre composites were performed using a Zwick Roell strength testing machine with a loading range of 0 to 10 kN. When testing fibre-cement samples, a constant increment in the crossbar movement was set at 0.1 mm/min. The spacing of supports in the machine was 200 mm and the force was applied axially.

The measurements of the acoustic emission used two frequency sensors: VS30-SIC (Vallen Systeme GmbH, Wolfratshausen, Germany) with flat characteristics in a range of 25–80 kHz, and VS150-RIC (Vallen Systeme GmbH, Wolfratshausen, Germany), with a measuring range of 100–450 kHz and a peak frequency of 150 kHz, alongside a 28 V AE signal preamplifier operating in three ranges: 20, 40, and 60 dB. In the preamplifier, the AE signal from the sensor was amplified and transmitted to an AE processor, where preliminary filtration was performed in order to eliminate the acoustic background originating from the surroundings of the monitored element. Subsequently, the signal was transformed into digital form. Further processing of the digital data was carried out using AE signal analysing software: Vallen VisualAE and Vallen VisualClass. 

Sensors recording the signals were placed in close proximity to the supports. The indicated locations of the sensors were selected in view of the relatively small dimensions of the sample and to ensure repetitiveness of the provided results. In each measurement case, the surface of the sensors was covered with silicone gel in order to achieve a better coupling between these elements. Pilot tests confirmed the correctness of the registration of the signals with the method of installation of the AE sensor as described above. As a standard benchmark, the Hsu–Nielsen pencil test (fracturing of the lead core of a 2 H pencil) was used to verify the correct operation of the sensors and the apparatus.

The application of the acoustic emission method during the three-point flexural test of fibre-cement panels facilitated the evaluation of changes in the mechanical parameters of these composites by associating the frequency of AE signals with the destructive processes taking place in the material, which gradually proceeded during bending.

## 3. Results

During the three-point bending tests, various mechanisms of sample destruction were observed. Elements in the air-dry state, soaked in water, subjected to cyclic bath-drying, and cyclically frozen–thawed (research cases P_1_–P_9_) due to the presence of reinforcing fibres deteriorated due to exceeding tensile stresses (damage was associated with a decrease in loads without breaking the sample). In the case of samples set on fire and fired in the furnace (cases P_10_–P_14_), a brittle mechanism of destruction was observed, specifically high energy, sudden fracture, and breakage of the samples into two parts.

Following the completion of the tests, the frequency values of AE signals in the analysed time series were monitored for the tested elements. In addition, the maximum values of loading force *F* were analysed for each sample.

**Figure 2:**
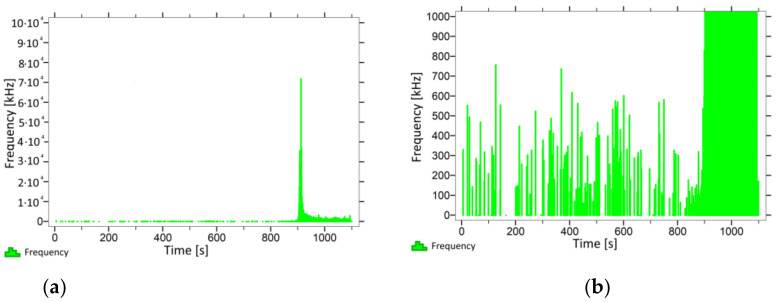
Graph of frequency distribution during the test for a representative sample from series P_1_: (**a**) considering the entire occurring frequency range and (**b**) with details about the frequency range before the moment of breakage.

**Figure 3:**
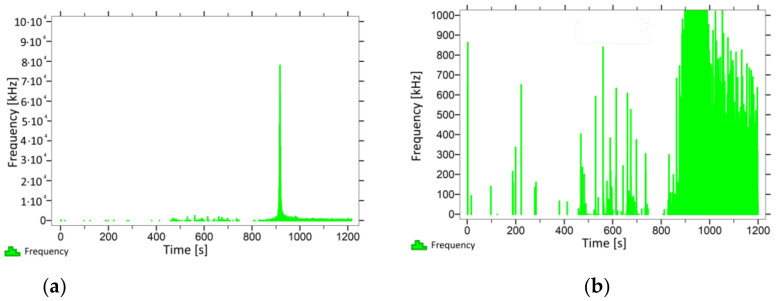
Graph of frequency distribution during the test for a representative sample from series P_2_: (**a**) considering the entire occurring frequency range and (**b**) with details about the frequency range before the moment of breakage.

**Figure 4:**
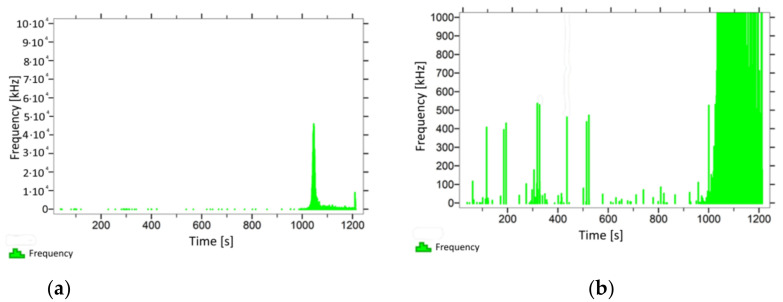
Graph of frequency distribution during the test for a representative sample from series P_3_: (**a**) considering the entire occurring frequency range and (**b**) with details about the frequency range before the moment of breakage.

**Figure 5:**
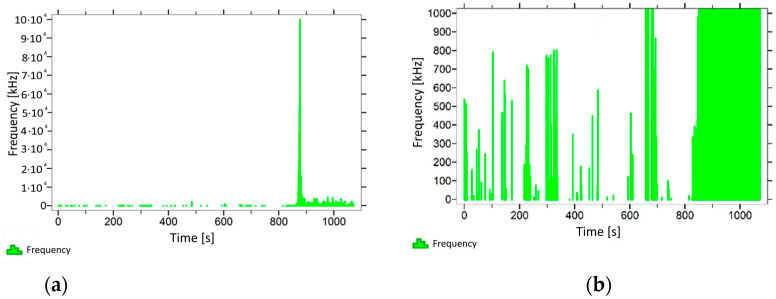
Graph of frequency distribution during the test for a representative sample from series P_4_: (**a**) considering the entire occurring frequency range and (**b**) with details about the frequency range before the moment of breakage.

**Figure 6:**
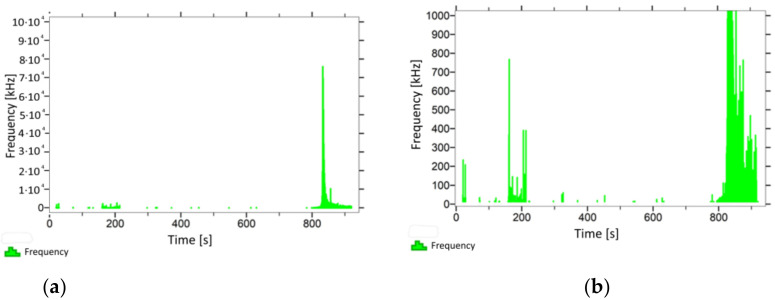
Graph of frequency distribution during the test for a representative sample from series P_5_: (**a**) considering the entire occurring frequency range and (**b**) with details about the frequency range before the moment of breakage.

**Figure 7:**
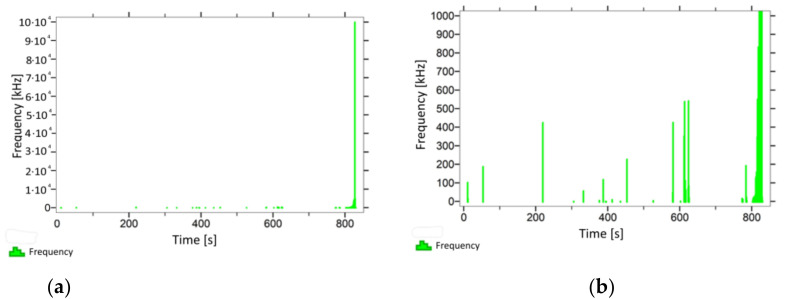
Graph of frequency distribution during the test for a representative sample from series P_6_: (**a**) considering the entire occurring frequency range and (**b**) with details about the frequency range before the moment of breakage.

**Figure 8:**
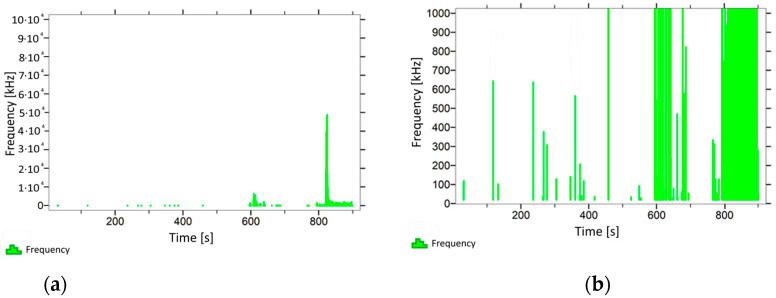
Graph of frequency distribution during the test for a representative sample from series P_7_: (**a**) considering the entire occurring frequency range and (**b**) with details about the frequency range before the moment of breakage.

**Figure 9:**
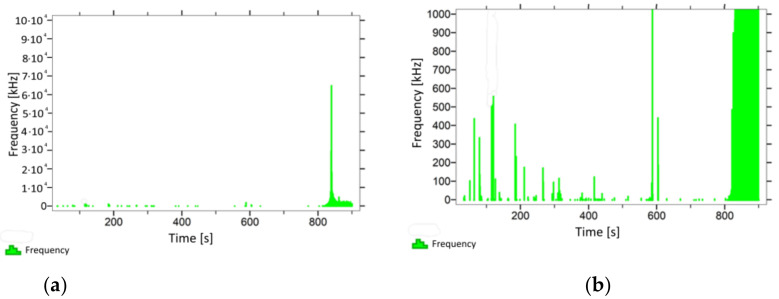
Graph of frequency distribution during the test for a representative sample from series P_8_: (**a**) considering the entire occurring frequency range and (**b**) with details about the frequency range before the moment of breakage.

**Figure 10:**
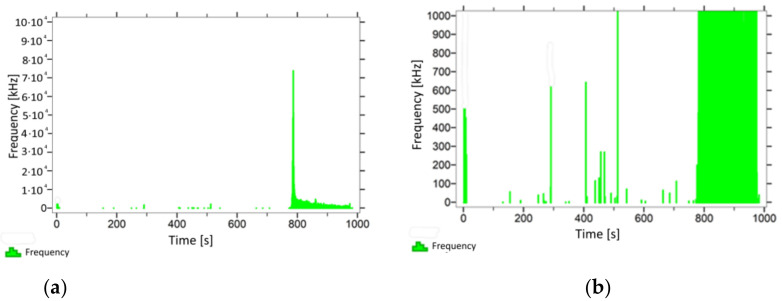
Graph of frequency distribution during the test for a representative sample from series P_9_: (**a**) considering the entire occurring frequency range and (**b**) with details about the frequency range before the moment of breakage.

With regard to samples from research cases P_1_–P_9_ (Figure 2, Figure 3, Figure 4, Figure 5, Figure 6, Figure 7, Figure 8, Figure 9 and Figure 10), the occurrence of the highest frequencies with values up to 5 × 10^4^–10 × 10^4^ kHz was recorded at the time of the sample-breaking. Before reaching the maximum stress, the frequencies in the range of 10–870 kHz were recorded.

**Figure 11:**
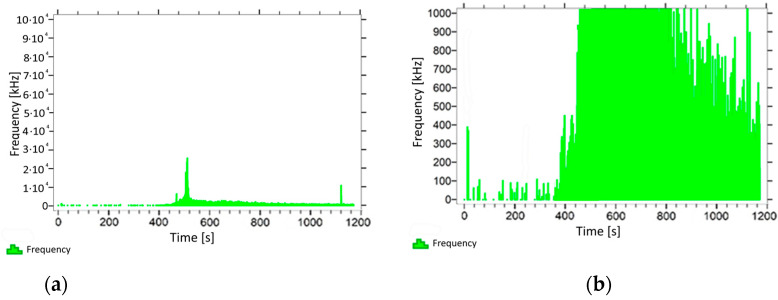
Graph of frequency distribution during the test for a representative sample from series P_10_: (**a**) considering the entire occurring frequency range and (**b**) with details about the frequency range before the moment of breakage.

With regard to the exemplary sample from the research case P_10_ (Figure 11), the occurrence of the highest frequencies with values up to 3 × 10^4^ kHz was recorded at the moment of the sample-breaking. Prior to reaching the maximum stress, frequencies mainly in the 5–400 kHz range were recorded.

**Figure 12:**
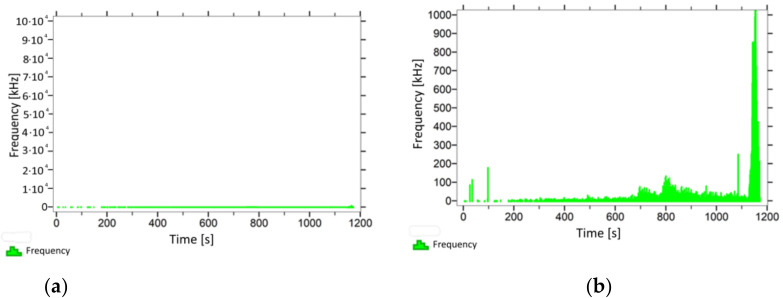
Graph of frequency distribution during the test for a representative sample from series P_11_: (**a**) considering the entire occurring frequency range and (**b**) with details about the frequency range before the moment of breakage.

**Figure 13:**
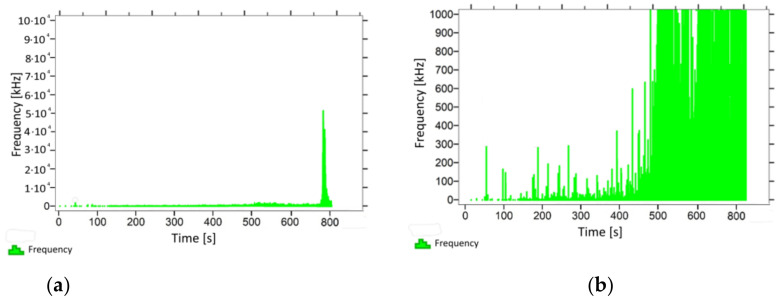
Graph of frequency distribution during the test for a representative sample from series P_12_: (**a**) considering the entire occurring frequency range and (**b**) with details about the frequency range before the moment of breakage.

**Figure 14:**
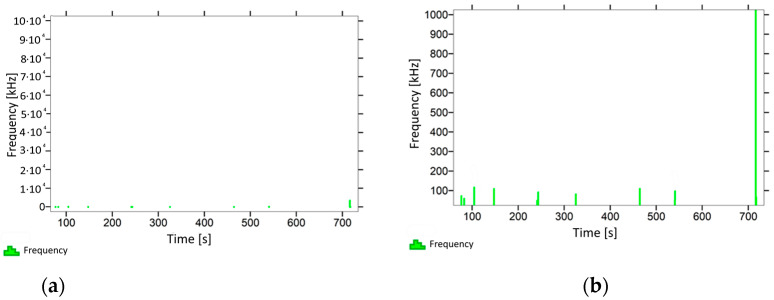
Graph of frequency distribution during the test for a representative sample from series P_13_: (**a**) considering the entire occurring frequency range and (**b**) with details about the frequency range before the moment of breakage.

**Figure 15:**
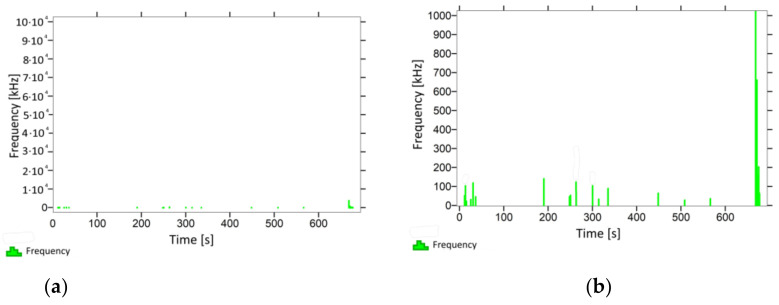
Graph of frequency distribution during the test for a representative sample from series P_14_: (**a**) considering the entire occurring frequency range and (**b**) with details about the frequency range before the moment of breakage.

With regard to the samples from test cases A_11_–P_14_ (Figure 12, Figure 13, Figure 14 and Figure 15), the frequencies did not exceed the value of 1 × 10^4^ kHz in the entire analysed waveform. Prior to reaching the maximum stresses, the frequencies mainly in the 5–190 kHz range were recorded.

### 3.1. Frequencies Analysis Results

### 3.2. Statistical Analysis of the Obtained Results

A statistical analysis of the obtained test results was performed in order to verify the usefulness of the analysis of the frequency of acoustic emission events for the purposes of the assessment of the deterioration of the mechanical parameters of cellulose–cement composites. During its first stage, this analysis involved a comparison of the results obtained for samples from the individual test cases with respect to specific quantitative variables. In the next step, the relationships between the indicated changes were examined (the significance of correlations was checked), followed by the use of classification trees utilising the CHAID algorithm to divide the results obtained for an analysed parameter within a given group and to determine any significant changes of these parameters. In order to confirm the possibility of using the frequencies accompanying changes in the mechanical parameters of cellulose–cement composites in the analysis, group-classifying data was used, with a subsequent performance of a test which compared the frequency distribution in the resulting groups in relation to the mechanical parameters of the samples.

The analysis utilised the IBM SPSS Statistics 26 software. The value of 0.05 was adopted as the significance level. The Shapiro–Wilk test was chosen for the analysis of the normality of distributions, while Levene’s test was used to examine the homogeneity of variances. Due to the absence of normal distribution for certain data and considering the lack of homogeneity of variances in most cases, a group of non-parametric tests for independent variables was used to mutually compare the average distributions, particularly the Kruskal–Wallis test for multiple groups.

At first, appropriate tests were performed for all data in order to select suitable groups of tests for the analysis of the data. The analysed groups were approximately equinumerous. Therefore, normal distributions of data in the individual groups were analysed using the Shapiro–Wilk test. In the case of most data, no reasons were found to reject the hypothesis of the normal distribution; however, there were cases in which the data did not have a normal distribution.

The absence of homogeneous variances was observed in most of the groups. Therefore, in order to analyse the distributions, a decision was made to use the non-parametric Kruskal–Wallis test for independent variables.

#### 3.2.1. Kruskal–Wallis Test Results for Independent Samples: Average Frequency of AE Events before Reaching *F_max_*

**Figure 16:**
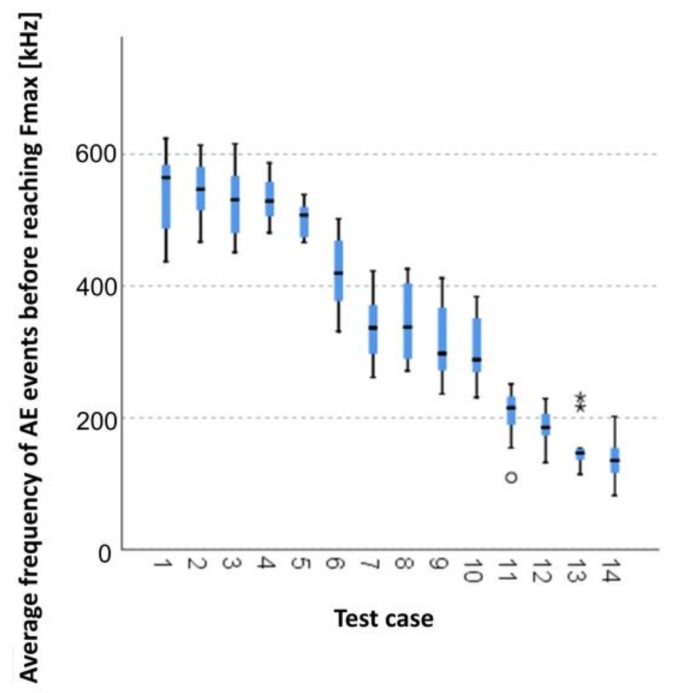
Graphic presentation of the Kruskal–Wallis test results for independent samples: average frequency of AE events before reaching *F_max_*.

When analysing the graphic presentation of the Kruskal–Wallis test results for independent samples, expressed by the average frequency of AE events before reaching *F_max_* (Figure 16), we could observe that the maximum average frequency of signals was recorded for elements of research case P_1_ (samples in an air-dry condition). Additionally, this case had the widest dispersion of results. Research cases P_11_ (samples ignited for 5 min) and P_13_ (samples ignited for 10 min) contained singular data which can be considered as statistical outliers. The lowest values of the average frequency of AE events before reaching Fmax were recorded for case P_14_ (the baked samples).

#### 3.2.2. Kruskal–Wallis Test Results for Independent Samples of the Breaking Force *F_max_*

**Figure 17:**
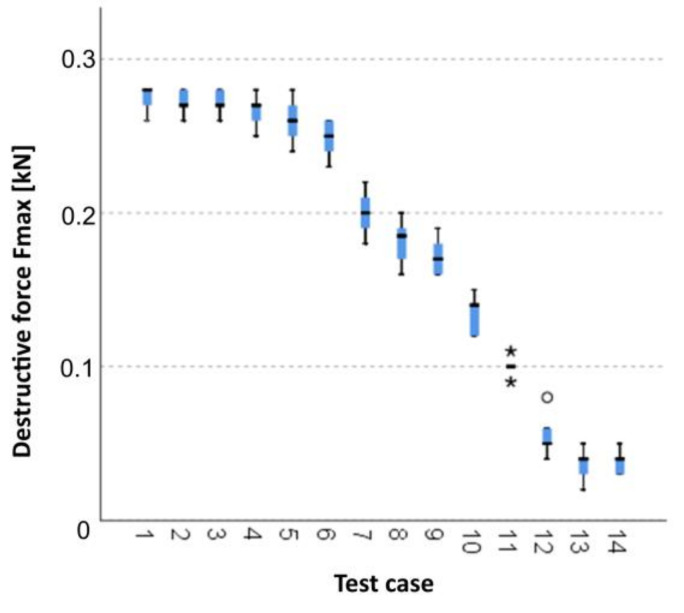
Graphic presentation of the Kruskal–Wallis test results for independent samples of the breaking force *F_max_*.

When analysing the graphic presentation of the Kruskal–Wallis test results for the independent testing of the breaking force *F_max_* (Figure 17), we could observe that the maximum breaking force was recorded for elements from research case P_5_ (samples subjected to bathing and drying in 50 cycles). Additionally, this case had the widest dispersion of results. Research cases P_11_ (samples ignited for 5 min) and P_12_ (samples ignited for 7.5 min) contained singular data which can be considered as statistical outliers. The lowest values of breaking force *F_max_* were recorded for case P_13_ (samples ignited for 10 min).

#### 3.2.3. Classification Trees

Classification trees utilising the CHAID algorithm were used in order to check the significance of the changes occurring in the parameters (frequency and breaking force).

**Figure 18:**
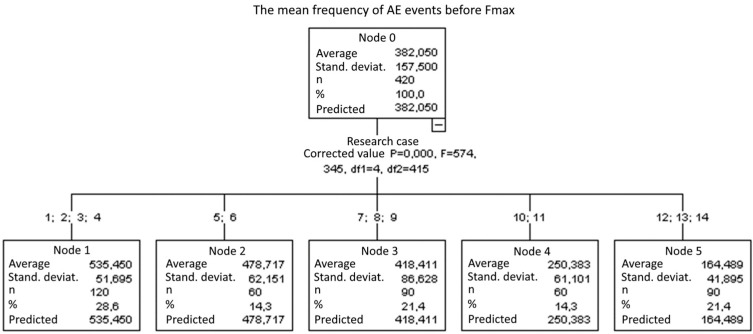
Classification tree for the average frequency of AE events before reaching *F_max_*.

**Figure 19:**
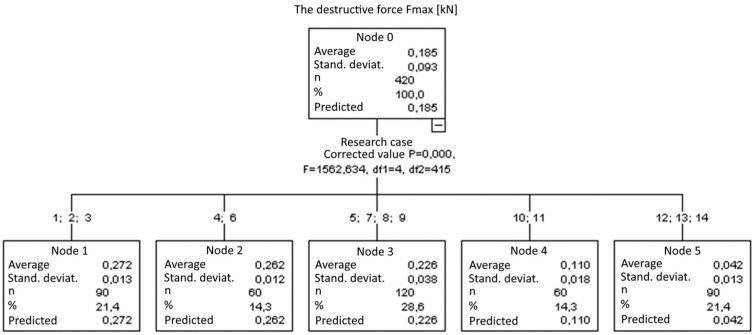
Classification tree for the breaking force *F_max_*.

1. Average frequency of AE events before reaching *F_max_*:

Five groups were identified (Figure 18). With each consecutive group, there was a significant reduction of the average frequency of AE events:

- Group 1, 2, 3, and 4;
- Group 5 and 6;
- Group 7, 8, and 9;
- Group 10 and 11; and
- Group 12, 13, and 14.

2. Breaking force *F_max_*:

Five groups were identified (Figure 19). With each consecutive group, there was a significant reduction of the *F_max_* breaking force:

- Group 1, 2, and 3;
- Group 4 and 6;
- Group 5, 7, 8, and 9;
- Group 10 and 11; and
- Group 12, 13, and 14.

#### 3.2.4. Kruskal–Wallis Test for Independent Samples: Average Frequency of AE Events before Reaching *F_max_* and the Breaking Force *F_max_*

Data used for the formation of groups in terms of changes in mechanical parameters obtained by means of classification trees was used to check the correlation of frequency changes with changes in mechanical parameters. An appropriate test that compared frequency distributions in these groups was selected: on each occasion, due to the absence of a normal distribution of frequencies in the individual groups, the non-parametric Kruskal–Wallis test was chosen. Mean values for each group were compared and, subsequently, confidence intervals were also assessed for the mean values (whether they overlapped each other). The Bonferroni test was used as a post-hoc test.

**Figure 20:**
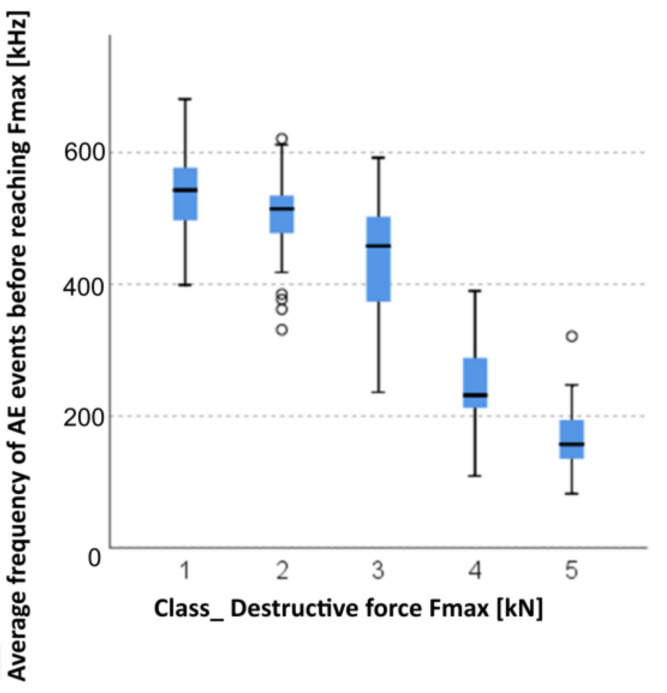
Graphic presentation of the Kruskal–Wallis test for independent samples: average frequency of AE events before reaching *F_max_* and the breaking force *F_max_*.

Five groups were identified (Figure 20). With each consecutive group, there was a significant reduction of the *F_max_* breaking force:

- Group 1: 1, 2, and 3;
- Group 2: 4 and 6;
- Group 3: 5, 7, 8, and 9;
- Group 4: 10 and 11; and
- Group 5: 12, 13, and 14.

The absence of a normal distribution was observed for the data. Therefore, the non-parametric Kruskal–Wallis test was chosen. At first, descriptive statistics were found for the groups. The Kruskal–Wallis test statistics T=327.370, p=0.000, and thus the frequencies in groups differed from each other in a statistically significant manner. The post-hoc Bonferroni test was performed in the second phase. In each case, between any two groups, the results differed from each other in a statistically significant manner. In each subsequent group, the frequencies were significantly lower (Figure 20). Moreover, we could observe that, although the frequency intervals overlappws each other (min/max), confidence intervals for the mean value did not overlap each other.

## 4. Discussion

When analysing the graphs shown in Figure 2, Figure 3, Figure 4, Figure 5, Figure 6, Figure 7, Figure 8, Figure 9, Figure 10, Figure 11, Figure 12, Figure 13, Figure 14 and Figure 15, we can observe that subjecting the tested elements to two groups of operating conditions (environmental and exceptional) resulted in significant differences in the emitted frequency ranges. Changes in the mechanical parameters of samples operating in an air-dry condition, saturated with water, subjected to cyclical baths and drying, as well as cyclically frozen and unfrozen during external loading are associated with the emission of low-frequency signals of up to 200 kHz and high-frequency signals of even up to 800 kHz. Most of the recorded frequencies exceeded the 200 kHz threshold and certain events generated sounds at a level of 500–800 kHz. An opposite situation occurred in the case of samples ignited for a time longer than 2.5 min or baked. The flexure of elements subjected to the impact of temperature caused events with considerably lower frequencies, only some of which exceeded a value of 100 kHz.

Based on the completed statistical analysis, it was demonstrated that some of the operating conditions, namely cyclical freezing–unfreezing, igniting with a flame, and baking at a temperature of 230 °C, which were applied to the cellulose–cement elements, have a significant effect on the change of the mechanical parameters. Conversely, the reduction in the strength of the panels was strictly related to a change in the acoustic characteristics registered during bending, in this case, identified with the average frequency of events before reaching *F_max_*. The performed statistical analysis allowed the authors to confirm the usefulness of the acoustic emission method in the assessment of changes in mechanical parameters of fibre-cement composites.

## 5. Conclusions

Considering the results of the tests and the performed statistical analysis, as well as the resultant preliminary conclusions indicating the usefulness of the acoustic emission method for the assessment of changes in the mechanical parameters of cellulose–cement composites, it was concluded that:

- an analysis of the frequency of AE events can be the basis for assessing the condition of cement–cellulose boards;
- an analysis of the obtained measurement results using the acoustic emission method enables the determination and assessment of the degree of changes in the mechanical parameters under the influence of the operational factors of the tested cement–cellulose boards;
- the intensity of changes taking place in the material and their impact on the strength parameters can be illustrated by using three terms referring to the condition of cement–cellulose elements, namely insignificant change, significant change, and critical change;
- a non-significant change in the mechanical parameters is associated with the emission of events before reaching the maximum load, with an average frequency above 300 kHz, while a significant change in mechanical parameters is identified with the average frequency of AE signals in the range of 200–300 kHz, and critical change in mechanical parameters has an average AE signal frequency of less than 200 kHz; and
- insignificant change in mechanical parameters is associated with a reduction of the bending strength by no more than 25% in relation to the reference panels, while a significant change is a reduction in strength by more than 25% but less than 50%, and the deterioration of strength properties by more than 50% is classified as a critical change in mechanical parameters.

The limits of the proposed method relate primarily to the limitations of the acoustic emission method itself. The main limitation is the ability to register only active processes in the material that lead to the release of elastic energy. Another disadvantage is the fact that the measurement enabling the frequency analysis to assess the condition of the cellulose–cement composite is longer compared to other diagnostic methods, e.g., the ultrasound method.

Further research, according to the authors, should concern the analysis of the frequency of events emitted by cellulose–cement composites with different fibre contents, as well as of the impact of UV radiation and an aggressive environment (related to the phenomenon of acid rain) on the mechanical parameters and sounds generated by the material.

## Figures and Tables

**Table 1:** The declared average technical parameters of the boards.

**Density**	Dry state	PN-EN 12467	≥1.58	g/cm^3^
**Flexural Strength**	Perpendicular	PN-EN 12467	25.0	N/mm^2^
**Flexural Strength**	In parallel	PN-EN 12467	18.0	N/mm^2^
**Modulus of Elasticity**		PN-EN 12467	12,000	N/mm^2^
**Stretching with Humidity**	30–95%		1.0	mm/m
**Porosity**	0–100%		<18	%

**Table 2:** Table of research cases of cellulose–cement composites with the adopted sample designation.

Case No.	Test Case	Designation
1.	Air-dry condition	P_1_
2.	Saturation with water for 1 h	P_2_
3.	Saturation with water for 24 h	P_3_
4.	25 bathing–drying cycles	P_4_
5.	50 bathing–drying cycles	P_5_
6.	10 freezing–unfreezing cycles	P_6_
7.	25 freezing–unfreezing cycles	P_7_
8.	50 freezing–unfreezing cycles	P_8_
9.	100 freezing–unfreezing cycles	P_9_
10.	Direct contact with a flame for 2.5 min	P_10_
11.	Direct contact with a flame for 5 min	P_11_
12.	Direct contact with a flame for 7.5 min	P_12_
13.	Direct contact with a flame for 10 min	P_13_
14.	Contact with the temperature of 230 °C for 3 h	P_14_

## Data Availability

Not applicable.
